# Carotenoid-Derived Flavor Precursors from *Averrhoa carambola* Fresh Fruit

**DOI:** 10.3390/molecules24020256

**Published:** 2019-01-11

**Authors:** Xuchao Jia, Dan Yang, Yue Yang, Haihui Xie

**Affiliations:** 1 Sericultural & Agri-Food Research Institute, Guangdong Academy of Agricultural Sciences/Key Laboratory of Functional Foods, Ministry of Agriculture and Rural Affairs/Guangdong Key Laboratory of Agricultural Products Processing, Guangzhou 510610, China; jiaxuchao@gdaas.cn; 2 South China Botanical Garden, Chinese Academy of Sciences/Guangdong Provincial Key Laboratory of Applied Botany/Key Laboratory of South China Agricultural Plant Molecular Analysis and Genetic Improvement, Guangzhou 510650, China; yangdan89@mail.jnmc.edu.cn (D.Y.); yangyue115@scbg.ac.cn (Y.Y.); 3 School of Public Health, Jining Medical University, Jining 272067, China; 4 College of Life Sciences, University of Chinese Academy of Sciences, Beijing 100049, China

**Keywords:** *Averrhoa carambola*, star fruit, flavor precursor, C_13_-norisoprenoid glucoside, C_15_ norisoprenoid

## Abstract

The fruit of *Averrhoa carambola* L. (Oxalidaceae), commonly known as star fruit or carambola, is popular in tropical and subtropical regions. Carotenoid-derived components, mainly C_13_- and C_15_-norisoprenoids, contribute greatly to the flavor of star fruit. Previously reported norisoprenoids were tentatively identified by GC-MS analysis after enzymatic hydrolysis. To gain accurate information about glycosidically bound flavor precursors in star fruit, a phytochemical study was conducted, which led to the isolation of 16 carotenoid derivatives—One new C_13_-norisoprenoid glucoside, (5*R*,6*S*,7*E*,9*R*)-5,6,9-trihydroxy-7-megastigmene 9-*O*-β-d-glucoside (**1**); one new C_15_-norisoprenoid, (6*S*,7*E*,10*S*)-Δ^9,15^-10-hydroxyabscisic alcohol (**11**); and 14 known ones, of which 12 were in glucoside form. The structures of the two new compounds were elucidated on the basis of extensive spectroscopic data analysis and chemical reaction. Compound **11** was a rare C_15_-norisoprenoid with a double bond between C-9 and C-15, and its possible biogenetic pathway was proposed. The known compounds were identified by comparison of their mass and nuclear magnetic resonance (NMR) data with those reported in the literature. The structure identification of one new (**1**) and seven known (**3**–**7**, **9**, and **10**) C_13_-norisoprenoid glucosides from the genus *Averrhoa* for the first time enriches the knowledge of carotenoid-derived flavor precursors in star fruit.

## 1. Introduction

**Figure 1:**
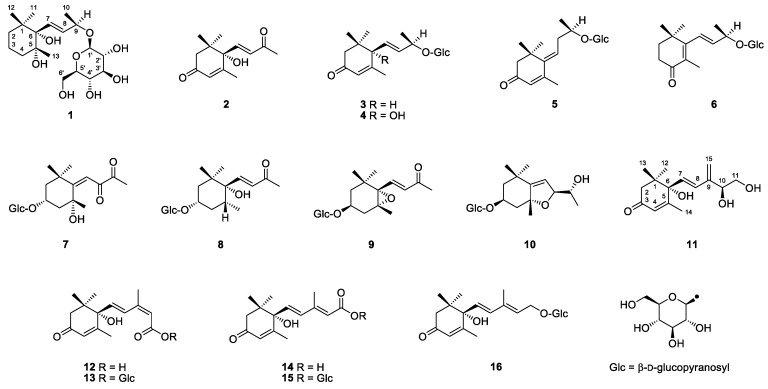
Chemical structures of compounds **1**–**16.**

*Averrhoa carambola* L., belonging to the family Oxalidaceae, is widely cultivated in Southeast Asia, China, and India. Its fruit, commonly known as star fruit or carambola, is popular in tropical and subtropical regions and consumed mostly as fresh fruit [1]. The volatile components of star fruit have been extensively studied and approximately 200 aroma components have previously been reported [2,3,4]. Volatile carotenoid breakdown products play an important role in the flavor of star fruit. However, β-ionone and β-ionol were detected as the only two C_13_-norisoprenoids from star fruit until MacLeod and Ames [4] reported 14 C_13_- and C_15_-aroma compounds by using the simultaneous distillation/extraction (SDE) method. Star fruit extract was subjected to SDE treatment and almond glucosidase hydrolysis by Herderich et al. [5], which liberated 29 C_13_-aroma compounds, indicating that these carotenoid-derived flavor components are derived from non-volatile flavorless glycosidic precursors and that vigorous isolation techniques such as SDE have a high probability of liberating C_13_-aroma components. The C_13_-norisoprenoid aroma components of star fruit have been well studied for their attractive sensory qualities and low flavor thresholds. However, the structural information about their glycosidically bound precursors is deficient, and hitherto only three ionone peracetylated glycosides have been isolated from star fruit and structurally identified by mass and NMR measurements [6,7]. Therefore, there is a need to study the accurate structural information of the precursors of these carotenoid-derived components. In the present study, 10 C_13_- and 6 C_15_-norisoprenoid compounds including two new ones (**1** and **11**) were isolated from fresh star fruit (Figure 1), and their structures were elucidated by spectroscopic methods. These findings enrich the knowledge of carotenoid-derived flavor precursors in star fruit.

## 2. Results and Discussion

**Figure 2:**
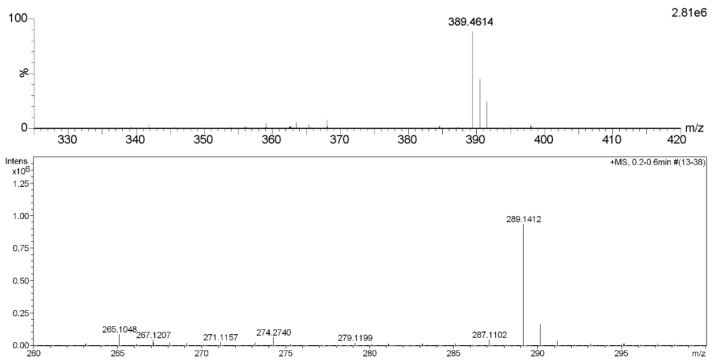
HR-ESI-MS spectra of compounds **1** (upper) and **11.**

**Figure 3:**
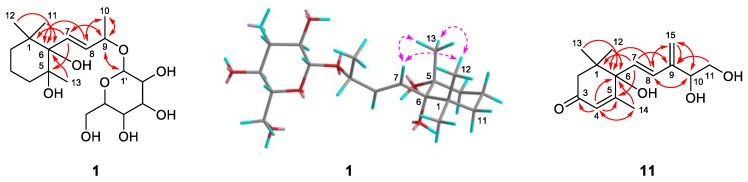
Key HMBC (plain) or NOESY (dashed) correlations of compounds **1** and **11.**

**Figure 4:**
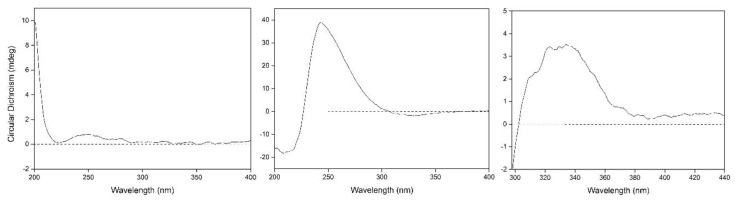
Circular dichroism (CD) spectra of compounds **1** (left) and **11** (middle) and induced circular dichroism (ICD) spectrum of **11** (right).

**Figure 5:**
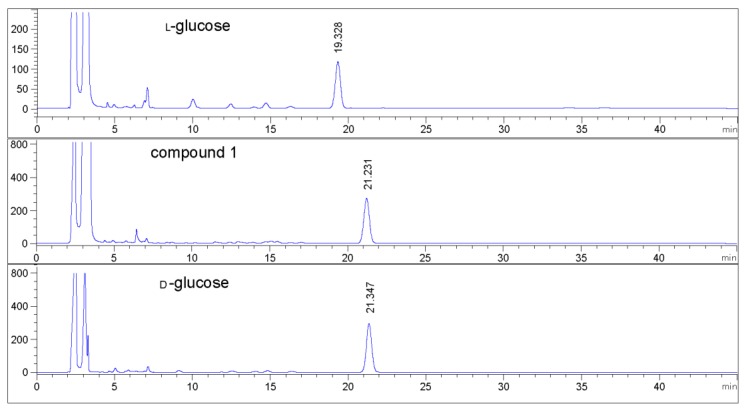
HPLC analytic spectra of glucosyl derivatives.

Compound **1** was obtained as colorless oil and deduced to have the molecular formula C_19_H_34_O_8_ based on the high-resolution electrospray ionization mass spectrometry (HR-ESI-MS) datum at *m/z* 389.4614 [M – H]^–^ (calculated for C_19_H_33_O_8_^−^, 389.4605) (Figure 2). The ^1^H and ^13^C NMR spectra (Table 1), coupled with heteronuclear single quantum coherence (HSQC) analysis, showed signals of four methyls, four methylenes, eight methines, and three quaternary carbons. The signals of δ_H_ 4.36 (1H, d, *J* = 7.8 Hz, H-1′) and δ_C_ 102.6 (C-1′), 75.4 (C-2′), 77.9 (C-3′), 71.5 (C-4′), 78.1 (C-5′), and 62.5 (C-6′) were typical of a β-glucosyl moiety. Apart from these signals, the remaining NMR data were in accordance with those of 7-megastigmene-5,6,9-triol [8] except for the remarkable downshift of C-9 at δ 78.4, which suggested that the β-glucosyl moiety was attached to C-9 [9]. This deduction was confirmed by the heteronuclear multiple bond correlation (HMBC) spectrum (Figure 3), in which the correlations from H-1′ to δ_C_ 78.4 (C-9) and δ_H_ 4.42 (H-9) to C-1′ were observed. In the nuclear overhauser effect spectroscopy (NOESY) spectrum (Figure 3), the presence of obvious correlations between δ_H_ 1.20 (3H, s, H_3_-13) and 6.15 (1H, d, *J* = 15.9 Hz, H-7), H-7 and δ_H_ 1.08 (3H, s, H_3_-12), H_3_-12 and H_3_-13 suggested that H-7, H_3_-12, and H_3_-13 were in the same orientation. Since the circular dichroism (CD) spectrum (Figure 4) showed a positive Cotton effect at 243 nm, C-6 was determined to have an *S* absolute configuration [10]. The absolute configuration of C-9 was determined as *R* based on the empirical rule summarized by Matsunami et al., in which the *δ* values (in CD_3_OD) of C-9, C-10, and C-1′ ranged from 77.3–79.1, 21.2–21.8, and 102.2–103.0 in 9*R* versus 74.7–76.3, 22.3–22.6, and 100.5–101.7 in 9*S* [11]. Acid hydrolysis of **1** yielded d-glucose (retention time (*t*_R_) = 21.2 min), which was ascertained by comparison of its *t*_R_ value with those of authentic d-glucose (*t*_R_ = 21.3 min) and l-glucose (*t*_R_ = 19.3 min) (Figure 5). Consequently, compound **1** was determined as (5*R*,6*S*,7*E*,9*R*)-5,6,9-trihydroxy-7-megastigmene 9-*O*-β-d-glucoside.

Compound **11** was acquired as a colorless oil with a positive optical rotation (+116.2 in MeOH). The molecular formula C_15_H_22_O_4_ was deduced from an HR-ESI-MS peak at *m*/*z* 289.1412 [M + Na]^+^ (calculated for C_15_H_22_NaO_4_^+^, 289.1410) (Figure 2), which requires five degrees of unsaturation. The ^1^H and ^13^C NMR data (Table 1) suggested that it was an abscisic alcohol derivative in comparison with those of abscisic alcohol β-d-glucoside (**16**) [12]. However, compound **11** showed the signals of two olefinic protons at δ_H_ 5.23 (1H, br s) and 5.33 (1H, br s), which were typical of a terminal double bond (H_2_-15). The HMBC correlations (Figure 3) from δ_H_ 5.99 (1H, d, *J* = 16.2 Hz, H-7) to δ_C_ 146.8 (C-9), H_2_-15 to δ_C_ 131.4 (C-8), 73.8 (C-10), and C-9 ascertained the terminal double bond between C-9 and C-15. In addition, the HMBC correlations from H-10 to C-8, δ_H_ 3.65 and 3.43 (1H each, dd, *J* = 11.4, 7.5 Hz, H_2_-11) to C-9 indicated a vicinal diol moiety connected to C-9. Further, the positive [α]_D_ value and positive Cotton effect at 243 nm in the CD spectrum (Figure 4) determined the *S* absolute configuration of C-6 [13,14]. In order to determine the absolute configuration of C-10, the induced circular dichroism (ICD) spectrum by Mo_2_(OAc)_4_ was measured using Snatzke′s method [15], which exhibited a positive Cotton effect at 310 nm after the addition of dimolybdenum tetraacetate in DMSO solution (Figure 4). According to the empirical rule, C-10 was determined to have an *S* absolute configuration. Therefore, compound **11** was identified as (6*S*,7*E*,10*S*)-Δ^9,15^-10-hydroxyabscisic alcohol.

**Figure 6:**
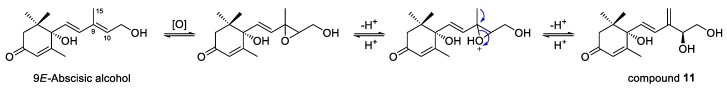
Possible biogenetic pathway of compound **11.**

The new compound **11** was a rare C_15_ carotenoid-derived norisoprenoid with a double bond between C-9 and C-15, and its possible biogenetic pathway was proposed, as shown in Figure 6. Compound **11** is likely generated from 9*E*-abscisic alcohol, the aglycone of compound **16**, by oxidation to form an epoxide between C-9 and C-10, and then dehydration to form a double bond between C-9 and C-15 and a hydroxyl group at C-10 under acidic conditions.

The 14 known compounds were determined as dehydrovomifoliol (**2**) [16], 3-oxo-*α*-ionol 9-*O*-β-d-glucoside (**3**) [17], roseoside (**4**) [17], 3-oxo-9-*O*-β-d-glucosyloxy-4,6*E*-megastigmadien (**5**) [18], 4-oxo-β-ionol 9-*O*-β-d-glucoside (**6**) [9], cannabiside D (**7**) [19], dendranthemoside B (**8**) [20], icariside B2 (**9**) [21], officinoside A (**10**) [22], abscisic acid (**12**) [23], abscisyl β-d-glucoside (**13**) [24], 9*E*-abscisic acid (**14**) [25], 9*E*-abscisyl β-d-glucoside (**15**) [26], and 9*E*-abscisic alcohol β-d-glucoside (**16**) [12] by comparing their spectroscopic data with those reported in the literature (see Supplementary Materials).

Glycosides perform accumulation, storage, and transport roles in aroma volatiles [27]. Herderich et al. [5] hydrolyzed star fruit extract with enzymes and identified 17 C_13_-norisoprenoids by GC-MS, including the aglycones of compounds **3**–**6**, **9**, and **10**. The aglycone of the new compound **1** was elucidated as tobacco′s flavor by Wahlberg [8]. Compound **2** was previously reported as the flavor of quince [28] and purple passion fruit [29], and this is the first time that it was characterized as star fruit′s fragrance. In addition to compound **2**, compounds **3**–**7**, **9**, and **10** were identified in the genus *Averrhoa* for the first time. The genin of compound **7** was not previously reported in nature. The peracetylated form of compound **8** was isolated from star fruit as an intact glycoconjugate flavor precursor [7].

## 3. Materials and Methods

### 3.1. General Experimental Procedures

ESI-MS spectra were measured on an MDS SCIEX API 2000 LC/MS/MS apparatus (Applied Biosystems Inc., Forster, CA, USA). The HR-ESI-MS spectrum of compound **1** was obtained on a Waters Xevo G2-XS QTOF mass spectrometer (Waters MS Technologies, Elstree, Hertfordshire, UK); a full MS scan was performed in the range of *m*/*z* 100–1500 Da, the capillary voltage was set at 2.5 kV, and the cone voltage was 40 V. Nitrogen gas was used for nebulizer and desolvation. The HR-ESI-MS spectrum of compound **11** was measured on a Bruker maXis mass spectrometer (Bruker Daltonics GmbH, Bremen, Germany); a full MS scan was performed in the range of *m*/*z* 100–2000 Da, the capillary voltage was set at 4.5 kV, and the end plate offset voltage was −500 V. One-dimensional (1D) and two-dimensional (2D) NMR spectra were recorded on a Bruker DRX-500 NMR spectrometer at 25 °C using solvent residual peaks as references. The ^1^H NMR spectra were run at 500.13 MHz proton frequency and the spectral width was 7500 Hz. The ^13^C NMR spectra were run at 125.77 MHz spectrometer frequency and the spectral width was 28,850 Hz. HSQC and HMBC experiments were measured using gradient selected sequences with 512 transients and 2048 data points for each of the 128 increments. The spectral widths were set at 5100 Hz for ^1^H and 27,500 Hz for ^13^C in the HSQC experiment, and 5100 Hz for ^1^H and 27,500 Hz for ^13^C in the HMBC experiment. For the NOESY experiment, 128 transients were collected into 1024 data points for each of the 160 increments with a spectral width of 3597 Hz for both dimensions. Optical rotation and ultraviolet (UV) spectra were acquired on a 343 polarimeter and a Lambda 650 UV/Vis spectrophotometer (Perkin-Elmer, Waltham, MA, USA), respectively. CD spectra were recorded on a Chirascan circular dichroism spectrometer (Applied Photophysics Ltd., Surrey, UK). Silica gel (100–200 mesh) was from Qingdao Haiyang Chemical Co. (Shandong, China), Amberlite XAD-7HP macroporous resin was from Sigma-Aldrich (St. Louis, MO, USA), and Sephadex LH-20 was from GE Healthcare Bio-Sciences AB (Uppsala, Sweden). Authentic d-(+)-glucose and l-(−)-glucose were from Aladdin Industrial Corp. (Shanghai, China). l-Cystein methyl ester hydrochloride was from Shanghai Macklin Biochemical Co. (Shanghai, China). *O*-Tolylisothiocyanate was from Tokyo Chemical Industry Co. (Tokyo, Japan). Thin layer chromatography (TLC) was conducted on pre-coated silica gel HSGF_254_ plates (Jiangyou Silica Gel Development Co., Yantai, China), and visualized by spraying 10% sulfuric acid in ethanol (*v*/*v*) followed by heating. Medium pressure liquid chromatography (MPLC) was performed on an EZ Purifier (Lisure Science, Suzhou, China) and the column used was a 400 mm × 25 mm inner diameter (i.d.) Chromatorex RP-18 SMB100, particle size 20–45 µm (Shanghai Lisui E-Tech Co., Shanghai, China). HPLC was conducted on a LC3000 set connected to a UV3000 scanning spectrophotometer detector (Beijing ChuangXin TongHeng Sci. and Tech. Co., Beijing, China) and the columns used were Cosmosil 5C18-MS-II (250 mm × 4.6 mm i.d., particle size 5 μm, Nacalai Tesque, Inc., Kyoto, Japan) for analysis and YMC-Pack ODS-A (250 mm × 20 mm i.d., particle size 5 μm, YMC Co., Kyoto, Japan) for preparation.

### 3.2. Plant Material

Fresh ripe star fruits (sweet in taste) were collected from an orchard (23°06′70.44″ N and 113°35′56.02″ E) in Xiaozhou Village, Haizhu District, Guangzhou in December of 2012.

### 3.3. Extraction and Isolation

The fresh fruits (105 kg) were cut to pieces and immediately soaked in 95% ethanol. The solution was filtrated after two days. The extraction steps were conducted three times. The combined solutions were concentrated under vacuum to give a brown syrup. The syrup was diluted with water and partitioned with ethyl acetate and then *n*-butanol. The solutions were evaporated to dryness to afford an ethyl acetate soluble fraction (77.4 g) and an *n*-butanol soluble fraction (590.2 g). The latter fraction was passed through an XAD-7HP macroporous resin column eluted with water and then 95% ethanol. The ethanol eluent was concentrated to dryness to result in an ethanol eluate (128.1 g). The eluate and ethyl acetate soluble fractions were combined and then subjected to silica gel column chromatography (CC) eluted with a chloroform/methanol gradient system (*v*/*v*, 1:0, 16 L; 95:5, 22.4 L; 9:1, 38.4 L; and 0:1, 16 L) to yield fractions 1–9 after being pooled according to their TLC profiles. Fraction 5 (19.0 g) was separated by silica gel CC eluted with a chloroform/methanol gradient system (*v*/*v*, 1:0, 6 L; 9:1, 7 L; 85:15, 6 L; 4:1, 8 L; 7:3, 6 L; 3:2, 6 L; and 1:1, 10 L) to provide fractions 5-1–5-7. Fraction 5-4 (65 mg) was separated by LH-20 CC eluted with methanol and then purified by HPLC using 62% methanol/water (*v*/*v*) as the mobile phase at a flow rate of 5 mL/min to yield compounds **14** (*t*_R_ = 22 min, 4 mg) and **12** (*t*_R_ = 25 min, 11 mg). Fraction 6 (6.0 g) was separated by MPLC using a methanol/water gradient system (*v*/*v*, 3:7, 2:3, 1:1, 3:2, 7:3, 4:1, and 1:0, each 2 L) as the mobile phase to yield fractions 6-1–6-6. Fraction 6-5 (27 mg) was purified by HPLC using 30% methanol/water (*v*/*v*) as the mobile phase at 5 mL/min to yield compound **11** (*t*_R_ = 51 min, 8 mg). Fraction 6-6 (75 mg) was separated by LH-20 CC eluted with methanol and then purified by HPLC using 30% methanol/water (*v*/*v*) as the mobile phase at 5 mL/min to result in compound **2** (*t*_R_ = 65 min, 9 mg). Fraction 8 (2.7 g) was separated by MPLC eluted with a methanol/water gradient system to provide fractions 8-1–8-10. Fraction 8-5 (250 mg) was separated by LH-20 CC eluted with methanol and then purified by HPLC using 12% methanol/water (*v*/*v*) as the mobile phase at 5 mL/min to yield compound **9** (*t*_R_ = 70 min, 4 mg). Fraction 8-9 (69 mg) was separated by LH-20 CC eluted with methanol and then purified by HPLC using 39% methanol/water (*v*/*v*) as the mobile phase at 5 mL/min to yield compounds **5** (*t*_R_ = 62 min, 10 mg) and **3** (*t*_R_ = 70 min, 5 mg). Fraction 8-10 (97 mg) was separated by LH-20 CC and then purified by HPLC using 39% methanol/water (*v*/*v*) as the mobile phase at 5 mL/min to yield compound **6** (*t*_R_ = 120 min, 9 mg). Fraction 9 (150.2 g) was subjected to silica gel CC eluted with a chloroform/methanol gradient system (*v*/*v*, 9:1, 15 L; 85:15, 23 L; 4:1, 20 L; 7:3, 38 L; 3:2, 40 L; and 1:1, 59 L) to provide fractions 9-1–9-8. Fraction 9-2 (1.25 g) was separated by MPLC to give fractions 9-2-1–9-2-17. Fraction 9-2-5 (56 mg) was purified by HPLC using 20% methanol/water (*v*/*v*) as the mobile phase at 5 mL/min to yield compounds **8** (*t*_R_ = 76 min, 5 mg) and **4** (*t*_R_ = 81 min, 15 mg). Fraction 9-2-8 (82 mg) was separated by LH-20 CC and then purified by HPLC using 14% acetonitrile/water (*v*/*v*) as the mobile phase at 6 mL/min to yield compounds **10** (*t*_R_ = 118 min, 6 mg), **15** (*t*_R_ = 129 min, 8 mg), and **13** (*t*_R_ = 139 min, 10 mg). Fraction 9-2-9 (120 mg) was separated by LH-20 CC and then purified by HPLC using 15% acetonitrile/water (*v*/*v*) as the mobile phase at 6 mL/min to result in compounds **1** (*t*_R_ = 61 min, 5 mg) and **16** (*t*_R_ = 64 min, 12 mg). Fraction 9-3 (6.13 g) was separated by MPLC to provide fractions 9-3-1–9-3-24. Fraction 9-3-12 (57 mg) was separated by LH-20 CC and then purified by HPLC using 15% acetonitrile/water (*v*/*v*) as the mobile phase at 6 mL/min to yield compound **7** (*t*_R_ = 61 min, 13 mg).

*(5R,6S,7E,9R)-5,6,9-Trihydroxy-7-megastigmene 9-O-β-d-glucoside* (**1**): Colorless oil; [*α*${]}_{D}^{20}$+24.7 (*c* 0.19, MeOH); CD (MeOH) ∆*ε* 247 (+0.97); HR-ESI-MS *m*/*z* 389.4614 [M – H]^−^ (calculated for C_19_H_33_O_8_^−^, 389.4605, error –2.3 ppm); ESI-MS *m*/*z* 413 [M + Na]^+^, 389 [M − H]^−^, and 425 [M + Cl]^−^; ^1^H NMR (500 MHz) and ^13^C NMR (125 MHz) data in CD_3_OD, see Table 1.

*(6S,7E,10S)-Δ^9,15^-10-Hydroxyabscisic alcohol* (**11**): Colorless oil; [*α*${]}_{D}^{20}$+116.2 (*c* 1.00, MeOH); UV (MeOH) λ_max_ nm (log *ε*) 230 (4.08); CD (MeOH) ∆*ε* 208 (–10.51), 243 (+22.43), and 329 (–1.06); HR-ESI-MS *m*/*z* 289.1412 [M + Na]^+^ (calculated for C_15_H_22_NaO_4_^+^, 289.1410, error −0.7 ppm); ESI-MS *m*/*z* 267 [M + H]^+^, 289 [M + Na]^+^, 265 [M − H]^−^, and 301 [M + Cl]^−^; ^1^H NMR (500 MHz) and ^13^C NMR (125 MHz) data in CD_3_OD, see Table 1.

### 3.4. Acid Hydrolysis

Acid hydrolysis was conducted following our previously reported procedures [30]. In brief, compound **1** (1 mg) was dissolved in 5 mL of 2 M aqueous hydrochloride and refluxed at 95 °C for 4 h. After removal of the solution under vacuum, 5 mL of water was added and partitioned with 5 mL of ethyl acetate three times. The aqueous layer was concentrated to dryness to yield a residue. The residue, authentic d-glucose, and l-glucose were individually dissolved in 1 mL of pyridine containing 1 mg/mL l-cystein methyl ester hydrochloride. After each solution was heated at 60 °C for 1 h, 2 mL of *O*-tolylisothiocyanate was added, heated at 60 °C for 1 h, and then concentrated to dryness. Each residue was dissolved in 1 mL of methanol and filtrated and analyzed by an Agilent Infinity 1260 HPLC at the wavelength of 254 nm and a 40 °C oven temperature. The column used was a Cosmosil 5C18-MS-II with acetonitrile/water/acetic acid (*v*/*v*, 22:78:0.1) as the mobile phase at a flow rate of 0.8 mL/min for 60 min, followed by washing with 90% acetonitrile/water (*v*/*v*).

### 3.5. Induced CD Spectrum by Mo_2_(OAc)_4_

According to the published procedure [15], about 1:1 diol-to-molybdenum mixtures were prepared using 0.66 mg/mL of compound **11**. The first CD spectrum was recorded soon after mixing and its evolution monitored for 30 min. The sign of the diagnostic band at 310 nm correlated with the absolute configuration of the diol moiety.

## 4. Conclusions

The fresh fruit of *Averrhoa carambola* (star fruit) possesses a fascinating and unique flavor, and carotenoid-derived C_13_- and C_15_-norisoprenoids contribute greatly to the flavor of star fruit. However, the exact structural information about the glycosidically bound precursors in star fruit was deficient. Our study on fresh star fruit led to the isolation of two new (**1** and **11**) and 14 known carotenoid-derived C_13_- and C_15_-norisoprenoids, of which 12 were in glucoside form. In addition to the two new compounds, compound **2** and seven known C_13_-norisoprenoid glucosides (**3**–**7**, **9**, and **10**) were identified from the genus *Averrhoa* for the first time. The new compound **11** was a rare C_15_ carotenoid-derived norisoprenoid with a terminal double bond between C-9 and C-15. In view of the previous reports of the aglycones of compounds **1**, **3**, **7**, and **8** and compound **2** itself as volatile flavor components in star fruit [6] or other fruits [8,28,29], it could be concluded that some of the 12 C_13_- and C_15_-norisoprenoid glucosides were carotenoid-derived flavor precursors in star fruit.

## Figures and Tables

**Table 1:** ^1^H and ^13^C NMR data of compounds **1** and **11** in CD_3_OD.

H/C	1		11	
	δ_H_ (mult, *J* in Hz)	δ_C_	δ_H_ (mult, *J* in Hz)	δ_C_
1		39.1, C		42.7
2	1.79 (td, 13.2, 3.7)	36.7, CH_2_	2.55 (d, 16.9)	50.7
	1.45 (m)		2.20 (d, 16.9)	
3	1.83 (m)	19.1, CH_2_		201.1
	1.34 (m)			
4	1.68 (td, 13.2, 3.7)	37.4, CH_2_	5.90 (br s)	127.3
	1.14 (m)			
5		75.7, C		167.0
6		79.9, C		80.3
7	6.15 (d, 15.9)	133.4, CH	5.99 (d, 16.2)	130.8
8	5.83 (dd, 15.9, 6.8)	133.7, CH	6.39 (d, 16.2)	131.4
9	4.44 (dq, 6.8, 6.3)	78.4, CH		146.8
10	1.34 (3H, d, 6.3)	21.6, CH	4.45 (t, 7.5)	73.8
11	0.84 (3H, s)	27.8, CH_3_	3.65 (dd, 11.4, 7.5)	67.3
			3.43 (dd, 11.4, 7.5)	
12	1.08 (3H, s)	27.1, CH_3_	1.05 (3H, s)	24.7
13	1.20 (3H, s)	25.7, CH_3_	1.02 (3H, d, 1.4)	23.5
14			1.92 (3H, br s)	19.5
15			5.33 (br s)	116.4
			5.23 (br s)	
1′	4.36 (d, 7.8)	102.6, CH		
2′	3.19 (dd, 9.2, 7.8)	75.4, CH		
3′	3.22 (m)	77.9, CH		
4′	3.32 (m)	71.5, CH		
5′	3.35 (m)	78.1, CH		
6′	3.82 (dd, 11.8, 2.4)	62.5, CH_2_		
	3.66 (dd, 11.8, 5.3)			

## Supplementary Materials

The following are available online. Figures S1–S5: 1D and 2D NMR spectra of compound **1**. Figure S6–S9: 1D and 2D NMR spectra of compound **11**. Spectroscopic data of 14 known compounds.
