# Supplementary material for: Carotenoid-Derived Flavor Precursors from Averrhoa carambola Fresh Fruit
Source: Molecules. 2019 Jan 11;24(2):256. doi: 10.3390/molecules24020256 (PMC6359364; doi:10.3390/molecules24020256)
Supplement: Supplementary file 1 [file molecules-24-00256-s001.pdf]

Supplementary Materials

Carotenoid-derived flavor precursors from *Averrhoa carambola* fresh fruit

Xuchao Jia, Dan Yang, Yue Yang, Haihui Xie

1. Figure captions

Figure S1. ^{1}H NMR spectrum of compound **1**

Figure S2. ^{13}C NMR spectrum of compound **1**

Figure S3. HSQC spectrum of compound **1**

Figure S4. HMBC spectrum of compound **1**

Figure S5. NOESY spectrum of compound **1**

Figure S6. ^{1}H NMR spectrum of compound **11**

Figure S7. ^{13}C NMR spectrum of compound **11**

Figure S8. HMBC spectrum of compound **11**

Figure S9. NOESY spectrum of compound **11**

2. Spectroscopic data of fourteen known compounds

Figure S1. ^{1}H NMR spectrum of compound **1**

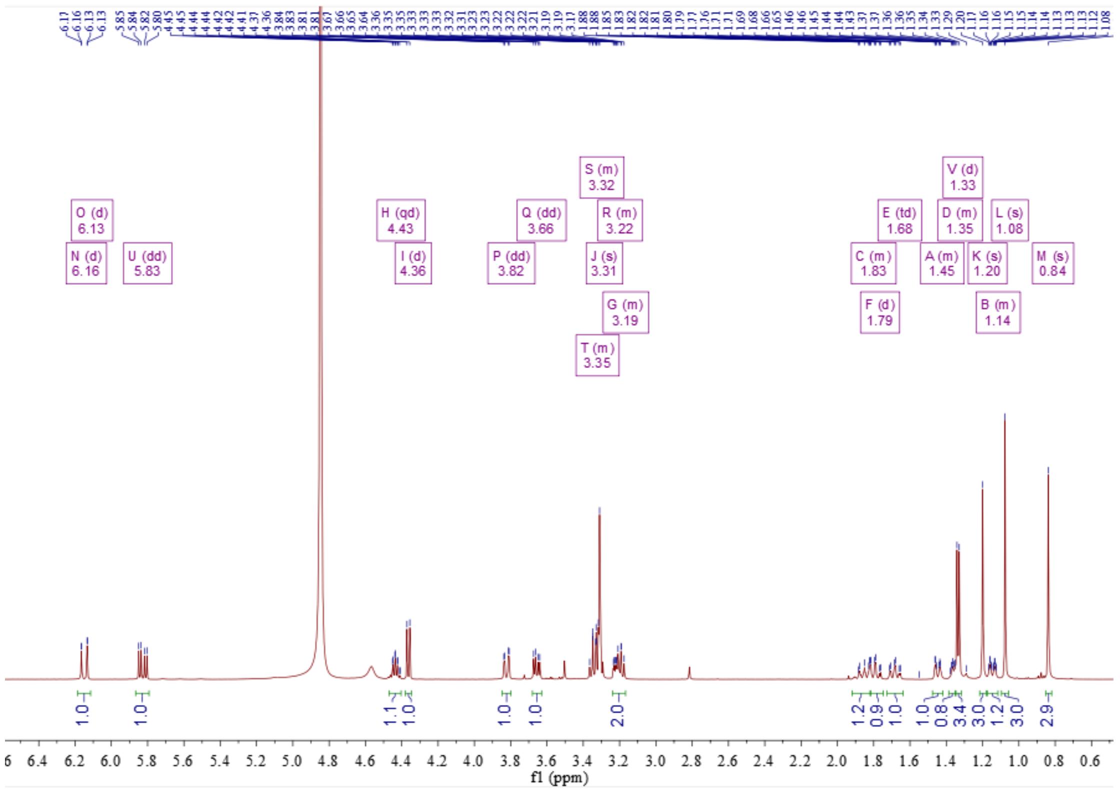

Figure S2. ^{13}C NMR spectrum of compound **1**

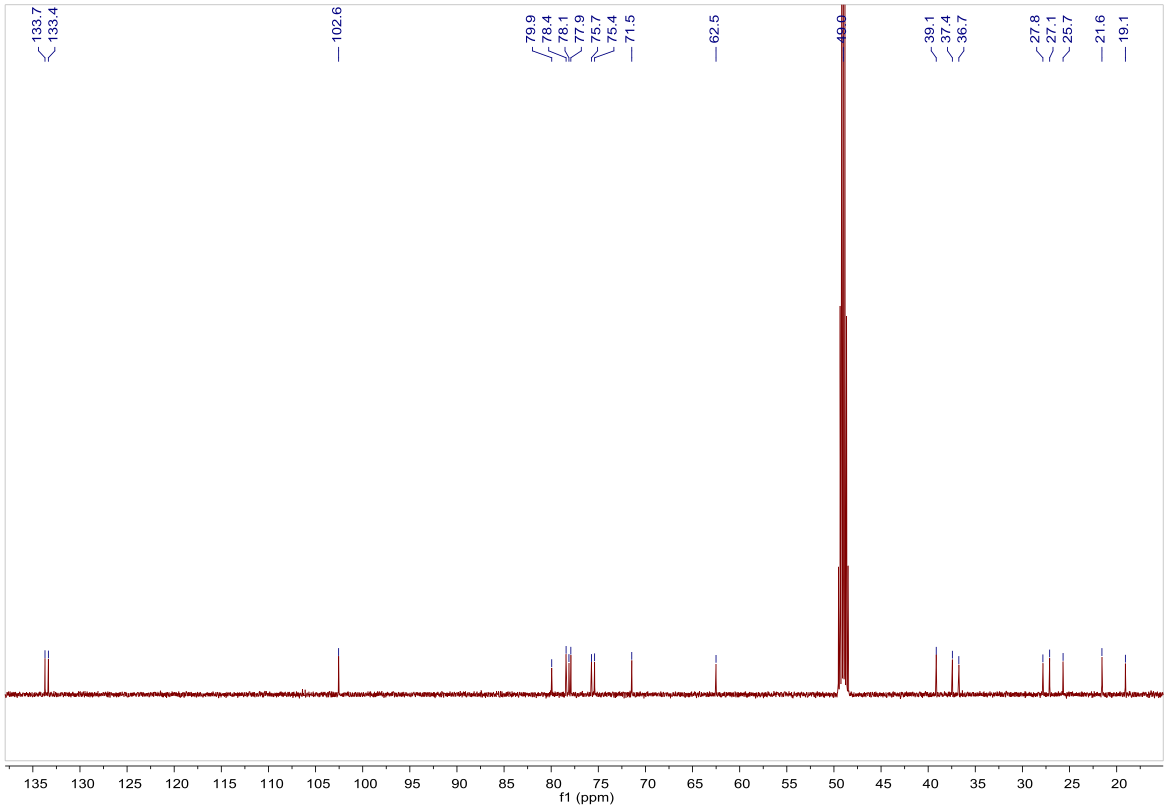

Figure S3. HSQC spectrum of compound **1**

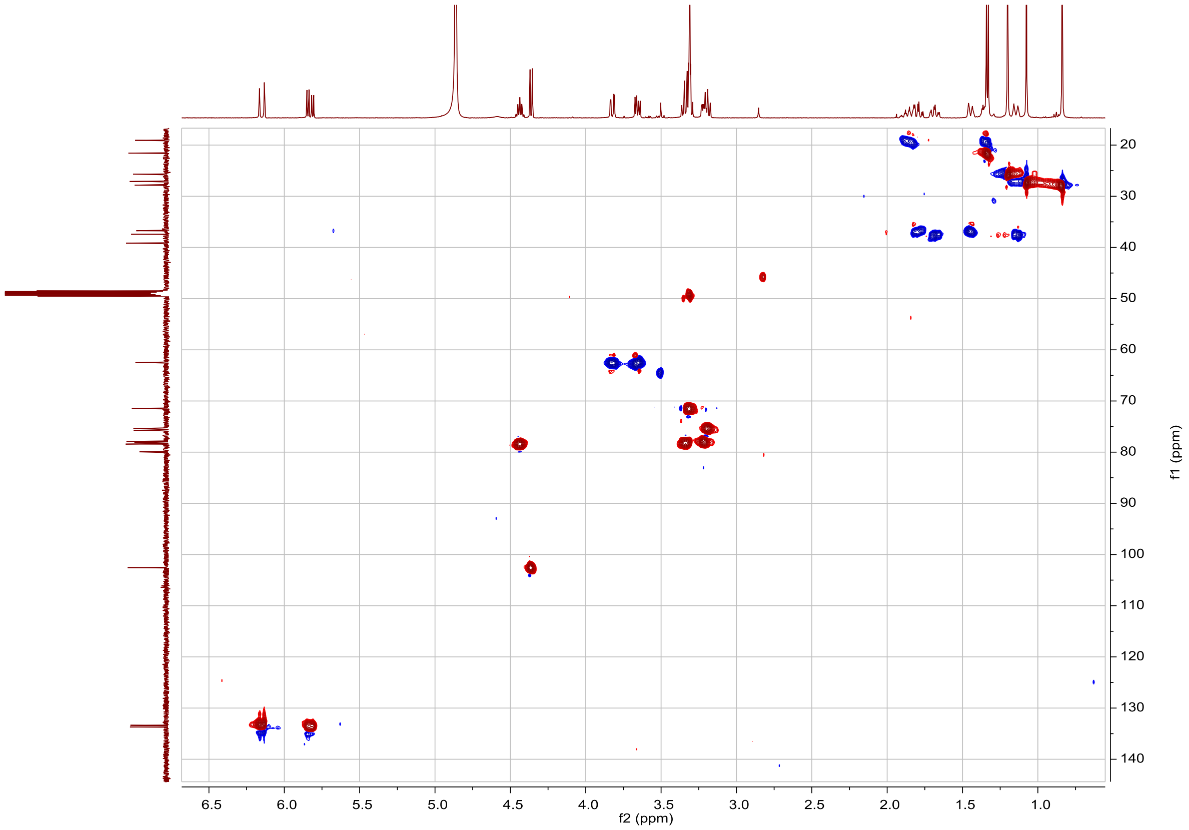

Figure S4. HMBC spectrum of compound **1**

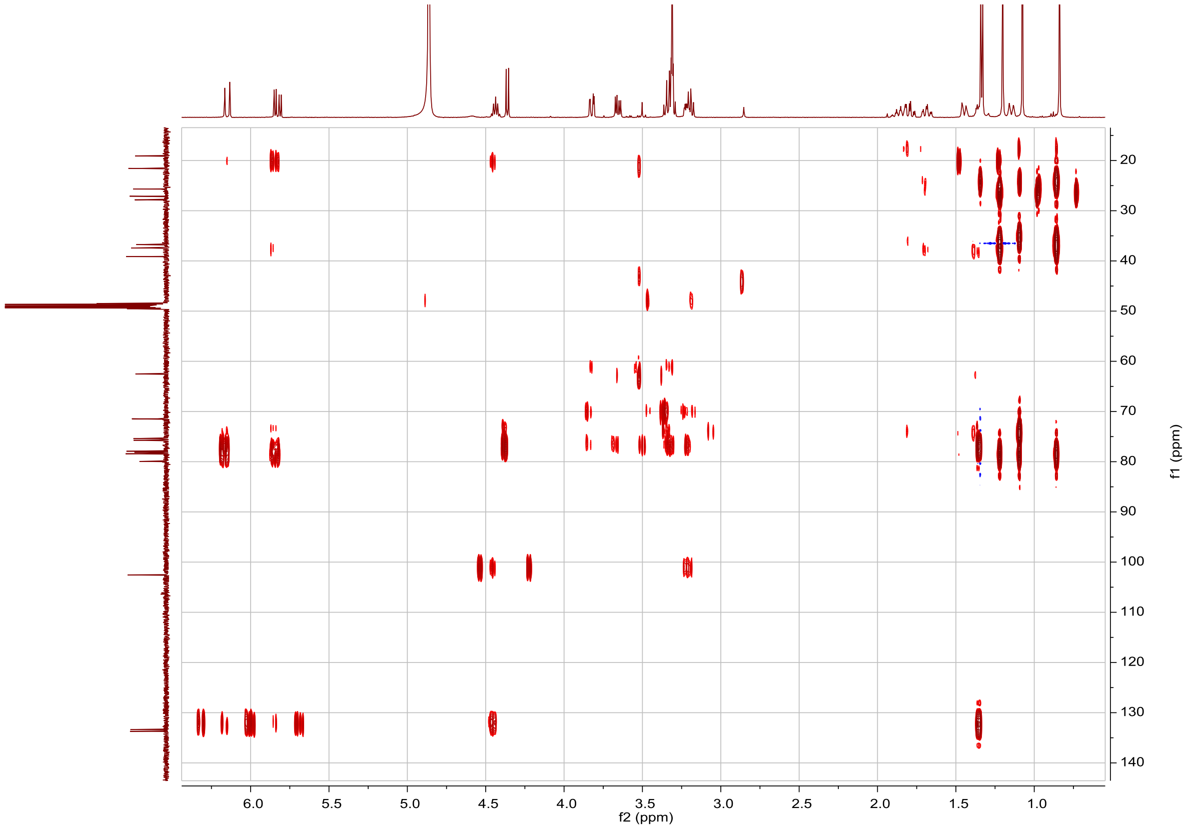

Figure S5. NOESY spectrum of compound **1**

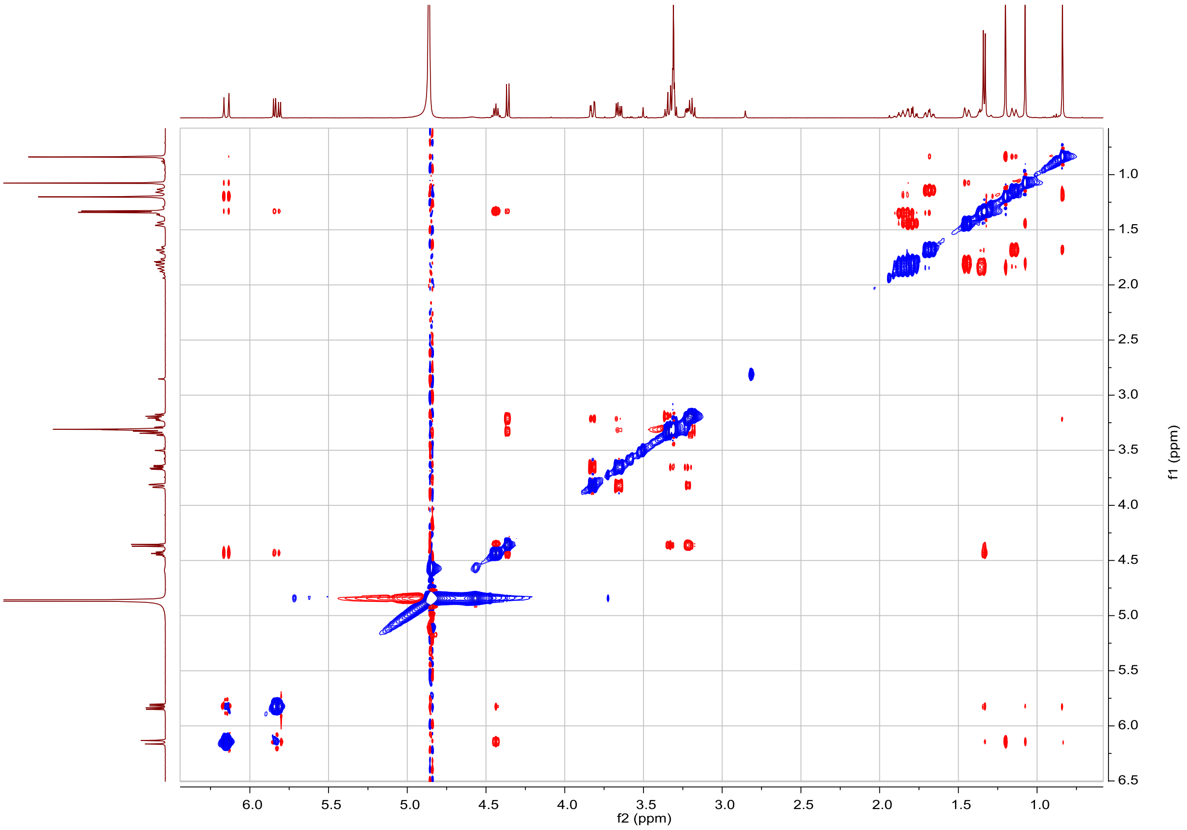

Figure S6. ^{1}H NMR spectrum of compound **11**

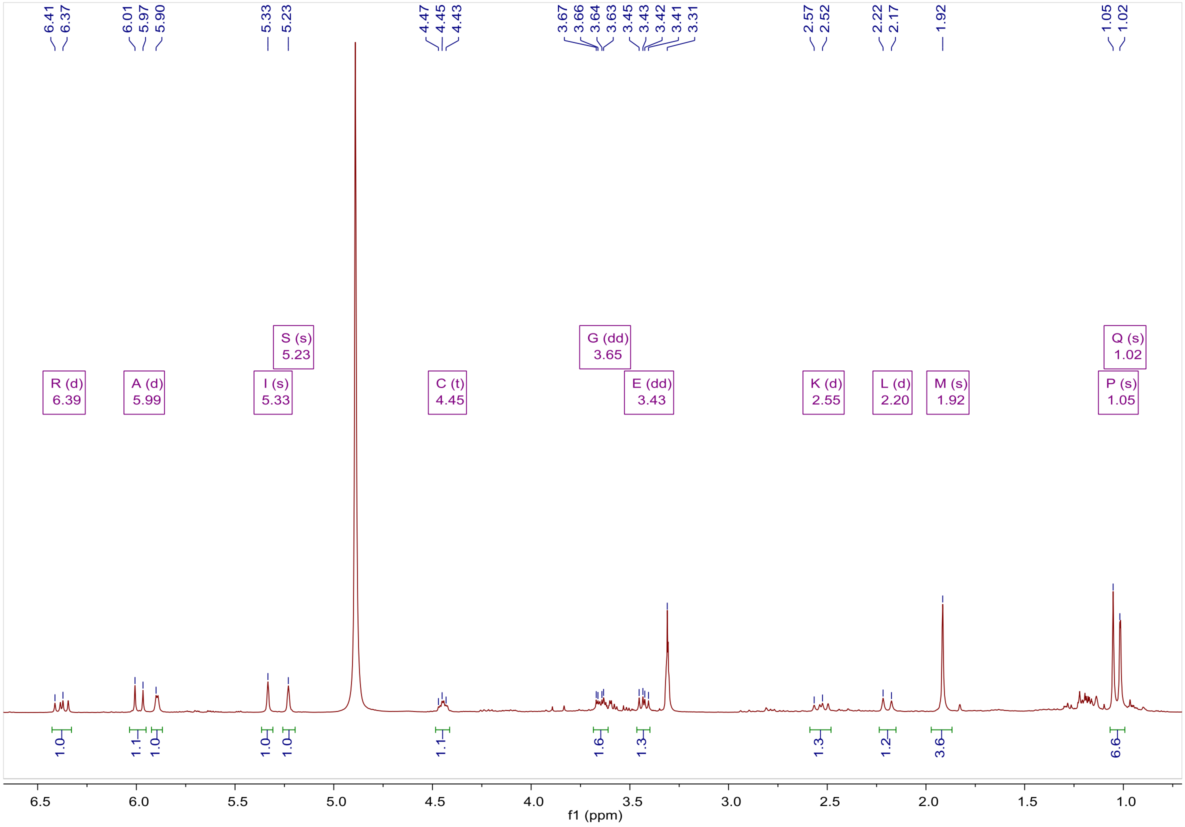

Figure S7. ^{13}C NMR spectrum of compound **11**

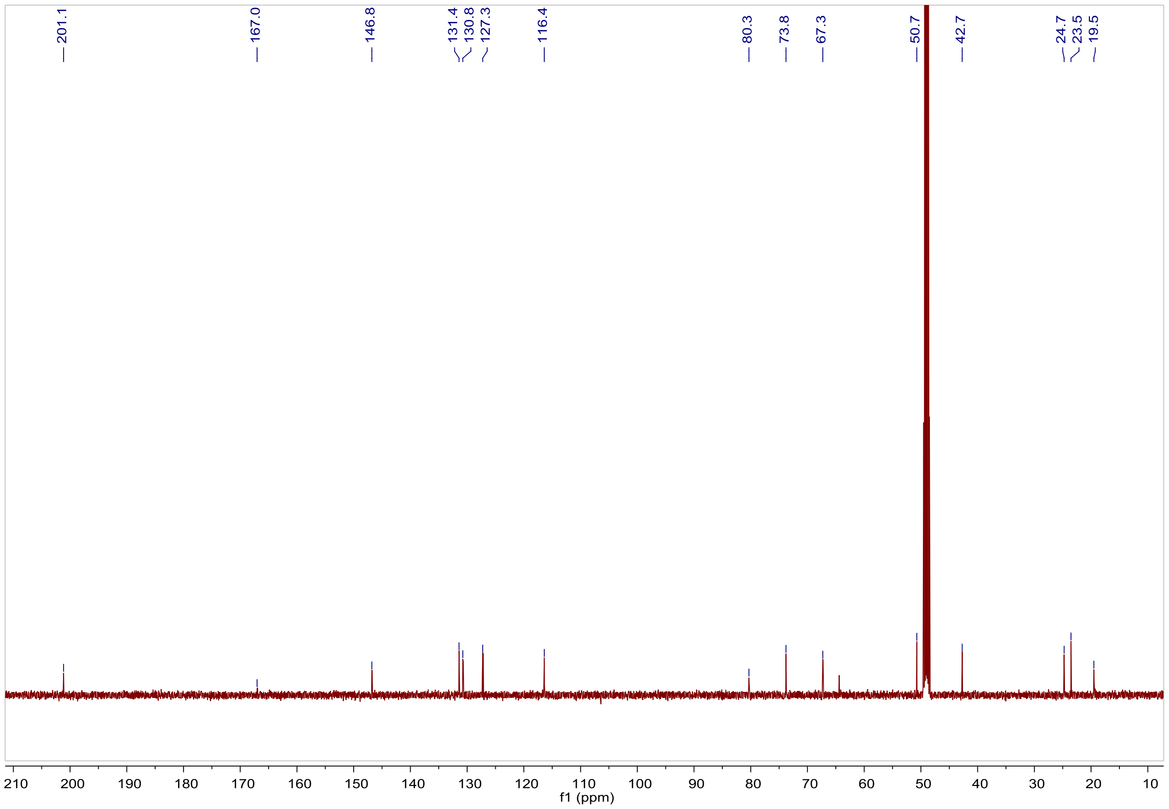

Figure S8. HMBC spectrum of compound **11**

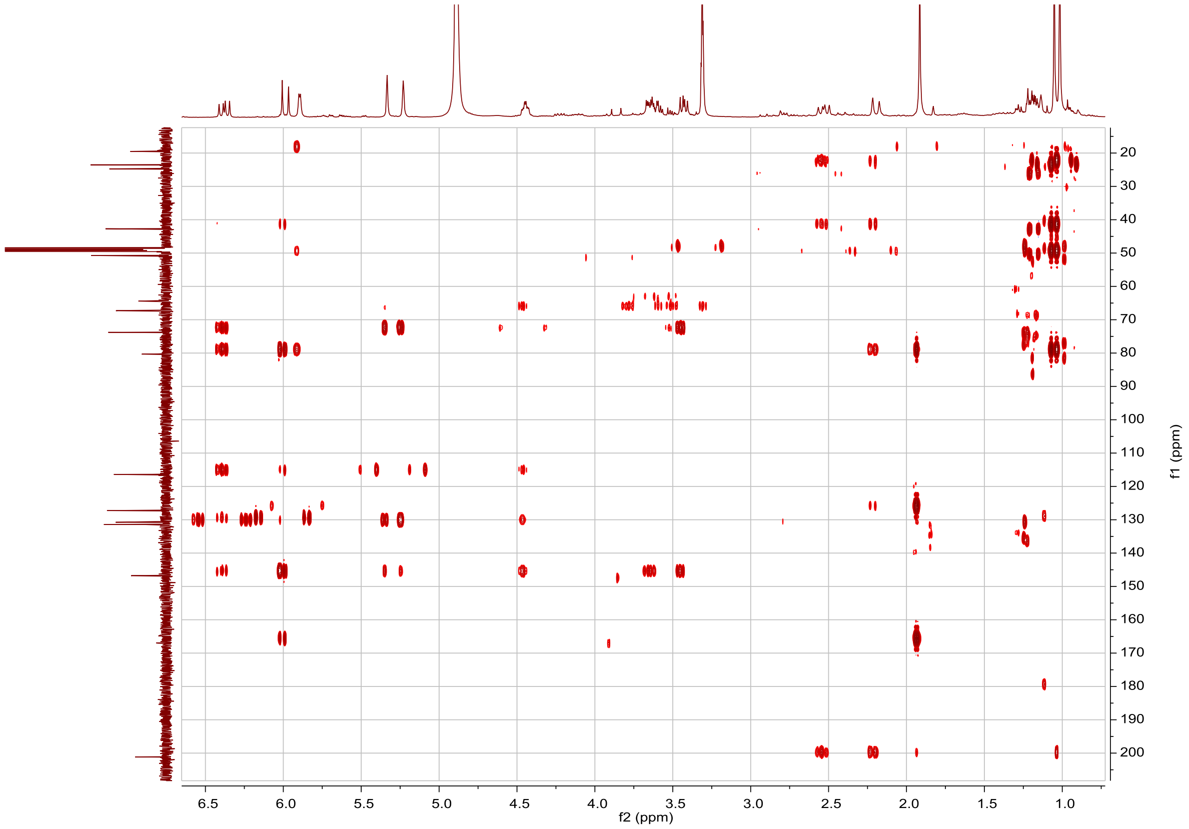

Figure S9. NOESY spectrum of compound **11**

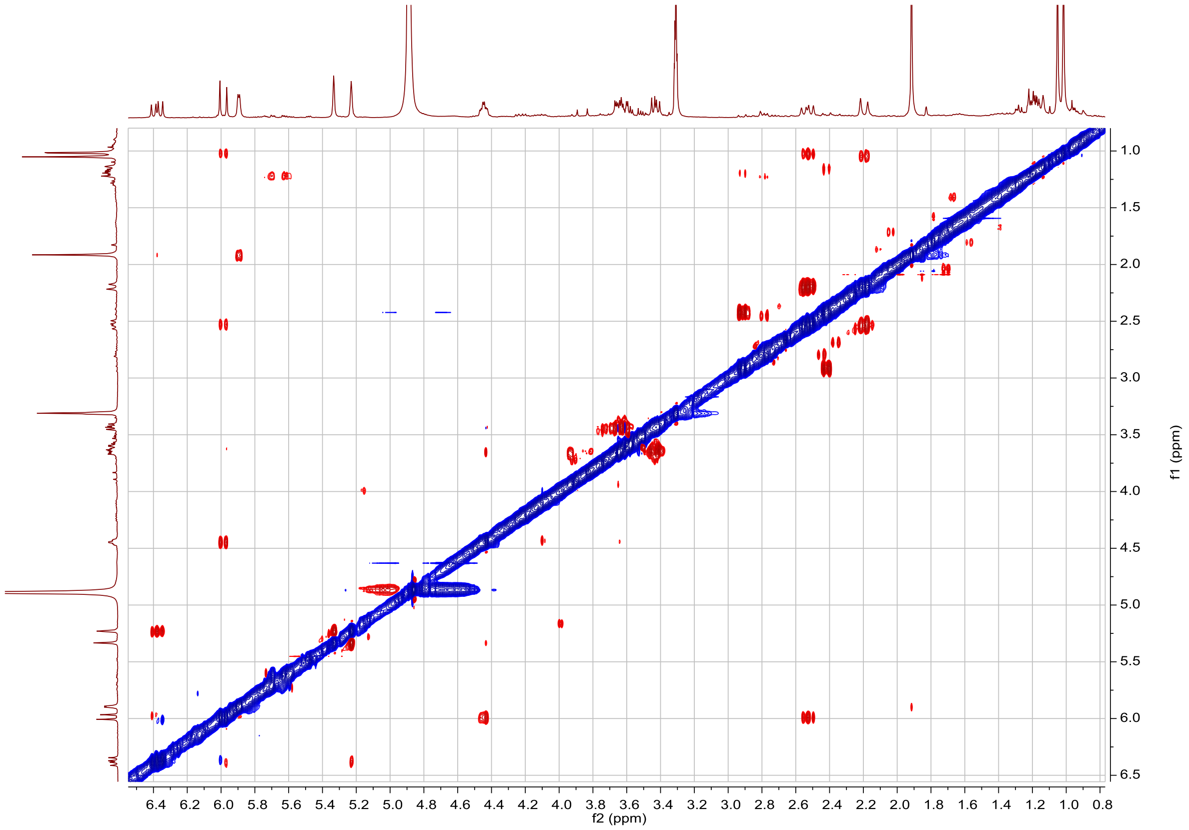

2. Spectroscopic data of fourteen known compounds

*2.1. Dehydrovomifoliol (**2**) [1]*

White amorphous powder; ESI-MS *m/z* 245 $[M + Na]^+$ and 257 $[M + Cl]^-$; ^1H NMR (400 MHz, CD_3OD) δ 2.61 (1H, d, *J* = 17.0 Hz, H-2), 2.28 (1H, d, *J* = 17.0 Hz, H-2), 5.94 (1H, s, H-4), 7.00 (1H, d, *J* = 15.8 Hz, H-7), 6.44 (1H, d, *J* = 15.8 Hz, H-8), 2.31 (3H, s, H_3-10), 1.01 (3H, s, H-11), 1.02 (3H, s, H-12), and 1.90 (3H, d, *J* = 1.2 Hz, H-13); ^{13}C NMR (100 MHz, CD_3OD) δ 42.6 (C-1), 50.5 (C-2), 200.4 (C-3), 128.0 (C-4), 164.7 (C-5), 80.0 (C-6), 148.3 (C-7), 131.7 (C-8), 200.7 (C-9), 27.6 (C-10), 23.5 (C-11), 24.7 (C-12), and 20.1 (C-13).

*2.2. 3-Oxo-α-ionol 9-O-β-D-glucoside (**3**) [2]*

White amorphous powder; ESI-MS *m/z* 371 $[M + H]^+$, 393 $[M + Na]^+$, 369 $[M - H]^-$, and 405 $[M + Cl]^-$; ^1H NMR (400 MHz, CD_3OD) δ 2.44 (1H, d, *J* = 16.8 Hz, H-2), 2.05 (1H, d, *J* = 16.8 Hz, H-2), 5.86 (1H, s, H-4), 2.68 (1H, d, *J* = 9.2 Hz, H-6), 5.65 (1H, dd, *J* = 15.5, 9.2 Hz, H-7), 5.78 (1H, dd, *J* = 15.5, 6.5 Hz, H-8), 4.40 (1H, dq, *J* = 6.5, 6.5 Hz, H-9), 1.29 (3H, d, *J* = 6.5 Hz, H_3-10), 1.03 (3H, s, H_3-11), 1.01 (3H, s, H_3-12), 1.94 (3H, d, *J* = 1.3 Hz, H_3-13), 4.34 (1H, d, *J* = 7.8 Hz, H-1'), 3.17 (1H, dd, *J* = 9.2, 7.8 Hz, H-2'), 3.26 (1H, t, *J* = 9.6 Hz, H-3'), 3.34 (1H, dd, *J* = 9.6, 8.9 Hz, H-4'), 3.22 (1H, m, H-5'), 3.82 (1H, dd, *J* = 11.8, 2.3 Hz, H-6'), and 3.66 (1H, dd, *J* = 11.8, 5.4 Hz, H-6'); ^{13}C NMR (100 MHz, CD_3OD) δ 37.1 (C-1), 48.3 (C-2), 202.0 (C-3), 126.1 (C-4), 165.9 (C-5), 56.8 (C-6), 128.8 (C-7), 138.2 (C-8), 77.0 (C-9), 21.0 (C-10), 28.0 (C-11), 27.6 (C-12), 23.8 (C-13), 102.4 (C-1'), 75.2 (C-2'), 78.0 (C-3'), 71.5 (C-4'), 78.1 (C-5'), and 62.7 (C-6').

*2.3. Roseoside (**4**) [2]*

White amorphous powder; ESI-MS *m/z* 387 [M + H]$^+$, 409 [M + Na]$^+$, 385 [M − H]$^-$, and 421 [M + Cl]$^-$; ^{1}H NMR (500 MHz, CD_3OD) *δ* 2.52 (1H, d, *J* = 17.0 Hz, H-2), 2.15 (1H, d, *J* = 17.0 Hz, H-2), 5.86 (1H, s, H-4), 5.88 (1H, d, *J* = 16.0 Hz, H-7), 5.84 (1H, dd, *J* = 16.0, 6.3 Hz, H-8), 4.42 (1H, m, H-9), 1.29 (3H, d, *J* = 6.4 Hz, H_3-10), 1.04 (3H, s, H_3-11), 1.03 (3H, s, H_3-12), 1.92 (3H, d, *J* = 1.2 Hz, H_3-13), 4.34 (1H, d, *J* = 7.8 Hz, H-1'), 3.17 (1H, dd, *J* = 9.2, 7.8 Hz, H-2'), 3.26 (1H, t, *J* = 9.6 Hz, H-3'), 3.34 (1H, dd, *J* = 9.6, 8.9 Hz, H-4'), 3.22 (1H, m, H-5'), 3.85 (1H, dd, *J* = 11.8, 2.0 Hz, H-6'), and 3.67 (1H, dd, *J* = 11.8, 5.4 Hz, H-6'); ^{13}C NMR (125 MHz, CD_3OD) *δ* 42.4 (C-1), 50.7 (C-2), 201.2 (C-3), 127.2 (C-4), 167.2 (C-5), 80.0 (C-6), 131.5 (C-7), 135.3 (C-8), 77.3 (C-9), 23.4 (C-10), 21.2 (C-11), 24.7 (C-12), 19.5 (C-13), 102.7 (C-1'), 75.2 (C-2'), 78.0 (C-3'), 71.6 (C-4'), 78.1 (C-5'), and 62.8 (C-6').

*2.4. 3-Oxo-9-O-β-D-glucosyloxy-4,6E-megastigmadien (**5**) [3]*

White amorphous powder; ESI-MS *m/z* 371 [M + H]$^+$, 393 [M + Na]$^+$, and 405 [M + Cl]$^-$; ^{1}H NMR (400 MHz, CD_3OD) *δ* 2.33 (2H, s, H_2-2), 5.87 (1H, br s, H-4), 6.36 (1H, t, *J* = 6.4 Hz, H-7), 2.68 (2H, m, H_2-8), 4.05 (1H, m, H-9), 1.26 (1H, d, *J* = 6.2 Hz, H-10), 1.30 (3H, s, CH_3-11), 1.29 (3H, s, CH_3-12), 2.13 (3H, br s, H_3-13), 4.36 (1H, d, *J* = 7.8 Hz, H-1'), 3.17 (1H, dd, *J* = 7.8, 9.2 Hz, H-2'), 3.89 (1H, dd, *J* = 11.7, 2.0 Hz, H-6), and 3.65 (1H, dd, *J* = 11.7, 5.7 Hz, H-6'); ^{13}C NMR (100 MHz, CD_3OD) *δ* 39.2 (C-1), 54.6 (C-2), 202.2 (C-3), 125.2 (C-4), 159.3 (C-5), 143.1 (C-6), 135.5 (C-7), 38.6 (C-8), 75.7 (C-9), 22.7 (C-10), 29.1 (C-11), 29.1 (C-12), 22.7 (C-13), 102.4 (C-1'), 75.1 (C-2'), 78.0 (C-3'), 71.9 (C-4'), 78.1 (C-5'), and 63.1 (C-6').

*2.5. 4-Oxo-β-ionol 9-O-β-D-glucoside (**6**) [4]*

White amorphous powder; ESI-MS *m/z* 393 [M + Na]$^+$ and 405 [M + Cl]$^-$; ^{1}H NMR (400 MHz, CD_3OD) *δ* 1.86 (2H, t, *J* = 6.8 Hz, H_2-2), 2.49 (2H, t, *J* = 6.8 Hz, H_2-3), 6.32 (1H, d, *J* = 16.2 Hz, H-7), 5.78 (1H, dd, *J* = 16.2, 6.3 Hz, H-8), 4.51 (1H, dq, *J* = 6.3, 6.3 Hz, H-9), 1.36 (3H, d, *J* = 6.3 Hz, H_3-10), 1.19 (3H, s, H_3-11), 1.18 (3H, s, H_3-12), 1.78 (3H, br s, H_3-13), 4.40 (1H, d, *J* = 7.8 Hz, H-1'), 3.20 (1H, dd, *J* = 9.0, 7.8 Hz, H-2'), 3.25 (1H, m, H-5'), 3.82 (1H, dd, *J* = 11.9, 2.4 Hz, H-6'), and 3.67 (1H, dd, *J* = 11.9, 5.0 Hz, H-6'); ^{13}C NMR (100 MHz, CD_3OD) *δ* 36.6 (C-1), 38.2 (C-2), 35.1 (C-3), 201.8 (C-4), 130.7 (C-5), 163.7 (C-6), 127.5 (C-7), 140.4 (C-8), 77.2 (C-9), 21.0 (C-10), 27.7 (C-11), 27.7 (C-12), 13.7 (C-13), 102.7 (C-1'), 75.3 (C-2'), 78.0 (C-3'), 71.5 (C-4'), 78.1 (C-5'), and 62.6 (C-6').

*2.6. Cannabiside D (**7**) [5]*

White amorphous powder; ESI-MS *m/z* 425 [M + Na]$^+$, 401 [M − H]$^-$, and 437 [M + Cl]$^-$; ^{1}H NMR (500 MHz, DMSO-d_6) *δ* 4.17 (1H, ddt, *J* = 11.4, 7.2, 4.1 Hz, H-3), 5.76 (1H, s, H-7), 2.11 (3H, s, H-10), 1.07 (3H, s, H-11), 1.32 (3H, s, H-12), 1.28 (3H, s, H-13), 4.24 (1H, d, *J* = 7.8 Hz, H-1'), 2.89 (1H, dd, *J* = 9.2, 7.8 Hz, H-2'), 3.14 (1H, t, *J* = 9.2 Hz, H-3'), 3.14 (1H, t, *J* = 9.2 Hz, H-4'), 3.10 (1H, ddd, *J* = 9.2, 5.5, 2.1 Hz, H-5'), 3.66 (1H, dd, *J* = 11.8, 2.1 Hz, H-6'), and 3.45 (1H, dd, *J* = 11.7, 5.5 Hz, H-6'); ^{13}C NMR (125 MHz, DMSO-d_6) *δ* 35.5 (C-1), 46.9 (C-2), 70.6 (C-3), 46.0 (C-4), 46.9 (C-5), 70.4 (C-6), 99.6 (C-7), 209.1 (C-8), 197.8 (C-9), 26.1 (C-10), 31.6 (C-11), 28.7 (C-12), 30.3 (C-13), 101.3 (C-1'), 73.5 (C-2'), 76.8 (C-3'), 70.0 (C-4'), 76.8 (C-5'), and 61.0 (C-6').

*2.7. Dendranthemoside B (**8**) [6]*

White amorphous powder; ESI-MS *m/z* 411 [M + Na]$^+$, 387 [M − H]$^-$, and 423 [M + Cl]$^-$; ^{1}H NMR (500 MHz, CD_3OD) *δ* 3.99 (1H, tt, *J* = 11.6, 4.7 Hz, H-3), 2.12 (1H, dqd, *J* = 13.4, 6.7, 3.5 Hz, H-5), 6.89 (1H, d, *J* = 16.0 Hz, H-7), 6.34 (1H, d, *J* = 16.0 Hz, H-8), 4.42 (1H, m, H-9), 2.28 (3H, s, H_3-10), 0.88 (3H, s, H_3-11), 1.05 (3H, s, H_3-12), and 0.81 (3H, d, *J* = 6.8 Hz, H_3-13), 4.37 (1H, d, *J* = 7.8 Hz, H-1'), 3.14 (1H,

dd, *J* = 9.2, 7.8 Hz, H-2'), 3.87 (1H, dd, *J* = 11.9, 1.7 Hz, H-6'), and 3.66 (1H, dd, *J* = 11.9, 5.7 Hz, H-6'); ^{13}C NMR (125 MHz, CD_3OD) δ 40.9 (C-1), 42.4 (C-2), 75.1 (C-3), 37.9 (C-4), 35.3 (C-5), 79.0 (C-6), 154.3 (C-7), 131.6 (C-8), 200.8 (C-9), 27.4 (C-10), 25.9 (C-11), 25.1 (C-12), 16.4 (C-13), 102.7 (C-1'), 75.4 (C-2'), 77.9 (C-3'), 71.7 (C-4'), 78.1 (C-5'), and 62.8 (C-6').

*2.8. Icariside B2 (**9**) [7]*

White amorphous powder; ESI-MS *m/z* 409 [M + Na]$^+$ and 421 [M + Cl]$^-$; ^{1}H NMR (500 MHz, CD_3OD) δ 3.92 (1H, dddd, *J* = 10.0, 8.5, 5.0, 3.4 Hz, H-3), 7.17 (1H, d, *J* = 15.8 Hz, H-7), 6.19 (1H, d, *J* = 15.8 Hz, H-8), 2.29 (3H, s, H-10), 1.21 (3H, s, H-11), 1.19 (3H, s, H-12), and 0.96 (3H, d, *J* = 6.8 Hz, H-13), 4.34 (1H, d, *J* = 7.8 Hz, H-1'), 3.12 (1H, dd, *J* = 9.0, 7.8 Hz, H-2'), 3.85 (1H, dd, *J* = 12.1, 1.9 Hz, H-6'), and 3.67 (1H, dd, *J* = 12.1, 5.4 Hz, H-6'); ^{13}C NMR (125 MHz, CD_3OD) δ 36.0 (C-1), 45.2 (C-2), 72.7 (C-3), 38.2 (C-4), 68.4 (C-5), 71.1 (C-6), 145.3 (C-7), 133.8 (C-8), 200.3 (C-9), 27.4 (C-10), 25.5 (C-11), 29.5 (C-12), 20.2 (C-13), 102.9 (C-1'), 75.1 (C-2'), 77.9 (C-3'), 71.6 (C-4'), 78.1 (C-5'), and 62.7 (C-6').

*2.9. Officinoside A (**10**) [8]*

White amorphous powder; ESI-MS *m/z* 411 [M + Na]$^+$, 387 [M − H]$^-$, and 423 [M + Cl]$^-$; ^{1}H NMR (500 MHz, CD_3OD) δ 2.00 (1H, ddd, *J* = 14.2, 3.6, 1.6 Hz, H-2), 1.46 (1H, dd, *J* = 14.2, 4.0 Hz, H-2), 4.20 (1H, m, H-3), 2.27 (1H, br d, *J* = 13.6 Hz, H-4), 1.70 (1H, dd, *J* = 13.6, 4.5 Hz, H-4), 5.53 (1H, d, *J* = 1.8 Hz, H-7), 4.33 (1H, dd, *J* = 6.3, 1.8 Hz, H-8), 3.55 (1H, dq, *J* = 6.3 Hz, H-9), 1.19 (3H, d, *J* = 6.3 Hz, H_3-10), 1.30 (3H, s, H_3-11), 1.19 (3H, s, H_3-12), 1.61 (3H, s, H_3-13), 4.34 (1H, d, *J* = 7.8 Hz, H-1'), 3.16 (1H, dd, *J* = 9.1, 7.8 Hz, H-2'), 3.28 (1H, t, *J* = 9.1 Hz, H-3'), 3.35 (1H, dd, *J* = 9.1, 8.2 Hz, H-4'), 3.25 (1H, m, H-5'), 3.86 (1H, dd, *J* = 11.9, 2.0 Hz, H-6'), and 3.66 (1H, dd, *J* = 11.9, 5.2 Hz, H-6'); ^{13}C NMR (125 MHz, CD_3OD) δ 35.1 (C-1), 46.8 (C-2), 75.2 (C-3), 44.4 (C-4), 88.9 (C-5), 155.1 (C-6), 118.6 (C-7), 89.1 (C-8), 71.8 (C-9), 19.9 (C-10), 28.4 (C-11), 31.7 (C-12), 31.2 (C-13), 102.8 (C-1'), 75.4 (C-2'), 77.9 (C-3'), 72.3 (C-4'), 78.5 (C-5'), and 62.9 (C-6').

*2.10. Abscisic acid (**12**) [9]*

White amorphous powder; ESI-MS *m/z* 287 [M + Na]$^+$, 263 [M − H]$^-$, 527 [2M− H]$^-$; ^{1}H NMR (CD_3OD, 500 MHz) δ 2.54 (1H, d, *J* = 16.9 Hz, H-2), 2.19 (1H, d, *J* = 16.9 Hz, H-2), 5.92 (1H, br s, H-4), 6.24 (1H, br d, *J* = 16.2 Hz, H-7), 7.78 (1H, dd, *J* = 16.2, 0.8 Hz, H-8), 5.75 (1H, s, H-10), 1.03 (3H, s, H-12), 1.07 (3H, s, H-13), 1.93 (3H, s, H-14), 2.04 (3H, s, H-15); ^{13}C NMR (CD_3OD, 125 MHz) δ 42.8 (C-1), 50.6 (C-2), 201.0 (C-3), 127.6 (C-4), 166.5 (C-5), 80.6 (C-6), 137.9 (C-7), 129.4 (C-8), 150.9 (C-9), 119.7 (C-10), 169.5 (C-11), 24.7 (C-12), 23.6 (C-13), 19.6 (C-14), 21.2 (C-15).

*2.11. Abscisyl β-D-glucoside (**13**) [10]*

White amorphous powder; ESI-MS *m/z* 449 [M + Na]$^+$, 425 [M − H]$^-$, 461 [M + Cl]$^-$; ^{1}H NMR (CD_3OD, 500 MHz) δ 2.54 (1H, d, *J* = 16.9 Hz, H-2), 2.20 (1H, d, *J* = 16.9 Hz, H-2), 5.94 (1H, br s, H-4), 6.33 (1H, dd, *J* = 16.1, 0.7 Hz, H-7), 7.81 (1H, dd, *J* = 16.1, 0.9 Hz, H-8), 5.82 (1H, br s, H-10), 1.07 (3H, s, H-12), 1.03 (3H, s, H-13), 1.93 (3H, d, *J* = 1.4 Hz, H-14), 2.08 (3H, d, *J* = 1.3 Hz, H-15), 5.50 (1H, d, *J* = 8.1 Hz, H-1'), 3.84 (1H, dd, *J* = 12.1, 2.1 Hz, H-6'), 3.68 (1H, dd, *J* = 12.1, 4.9 Hz, H-6'); ^{13}C NMR (CD_3OD, 125 MHz) δ 42.9 (C-1), 50.6 (C-2), 200.9 (C-3), 127.7 (C-4), 166.3 (C-5), 80.6 (C-6), 139.3 (C-7), 129.2 (C-8), 153.6 (C-9), 118.1 (C-10), 165.8 (C-11), 24.7 (C-12), 23.6 (C-13), 19.6 (C-14), 21.3 (C-15), 95.4 (C-1'), 74.0 (C-2'), 78.0 (C-3'), 71.1 (C-4'), 78.8 (C-5'), 62.3 (C-6').

*2.12. 9E-Abscisic acid (**14**) [11]*

White oil; ESI-MS *m/z* 287 [M + Na]$^+$, 263 [M − H]$^-$, and 527 [2M − H]$^-$; ^{1}H NMR (CD_3OD, 500 MHz) δ 2.55 (1H, d, *J* = 17.0 Hz, H-2), 2.23 (1H, d, *J* = 17.0 Hz, H-2), 5. 91 (1H, s, H-4), 6.25 (1H, d, *J* = 15.8 Hz, H-7), 6.47 (1H, d, *J* = 15.8 Hz, H-8), 5.84 (1H, s, H-10), 1.01 (3H, s, H_3-12), 1.06 (3H, s, H_3-13), 1.91 (3H, s, H_3-14), 2.26 (3H, s, H_3-15); ^{13}C NMR (CD_3OD, 125 MHz) δ 42.8 (C-1), 50.7 (C-2), 200.9 (C-3), 127.4 (C-4), 166.3 (C-5), 80.3 (C-6), 136.5 (C-7), 135.3 (C-8), 151.6 (C-9), 122.1 (C-10), 170.4 (C-11), 24.7 (C-12), 23.6 (C-13), 19.4 (C-14), 14.2 (C-15).

*2.13. 9E-Abscisyl β-D-glucoside (**15**) [12]*

White amorphous powder; ESI-MS *m/z* 449 [M + Na]$^+$, 425 [M − H]$^-$, 461 [M + Cl]$^-$; ^{1}H NMR (CD_3OD, 500 MHz) δ 2.56 (1H, d, *J* = 17.0 Hz, H-2), 2.24 (1H, d, *J* = 17.0 Hz, H-2), 5.92 (1H, br s, H-4), 6.36 (1H, d, *J* = 15.7 Hz, H-7), 6.53 (1H, dd, *J* = 15.7, 0.8 Hz, H-8), 5.91 (1H, br s, H-10), 1.06 (3H, s, H-12), 1.01 (3H, s, H-13), 1.90 (3H, d, *J* = 1.4 Hz, H-14), 2.32 (3H, d, *J* = 1.2 Hz, H-15), 5.52 (1H, d, *J* = 8.1 Hz, H-1'), 3.84 (1H, dd, *J* = 12.1, 2.1 Hz, H-6'), 3.68 (1H, dd, *J* = 12.1, 4.9 Hz, H-6'); ^{13}C NMR (CD_3OD, 125 MHz) δ 42.8 (C-1), 50.6 (C-2), 200.8 (C-3), 127.5 (C-4), 166.7 (C-5), 80.3 (C-6), 138.1 (C-7), 134.8 (C-8), 155.0 (C-9), 119.9 (C-10), 160.0 (C-11), 24.7 (C-12), 23.6 (C-13), 19.3 (C-14), 14.4 (C-15), 95.4 (C-1'), 74.0 (C-2'), 78.0 (C-3'), 71.1 (C-4'), 78.8 (C-5'), 62.3 (C-6').

*2.14. 9E-Abscisic alcohol β-D-glucoside (**16**) [13]*

White amorphous powder; ESI-MS *m/z* 435 [M + Na]$^+$, 411 [M − H]$^-$, and 447 [M + Cl]$^-$; ^{1}H NMR (CD_3OD, 500 MHz) δ 2.49 (1H, d, *J* = 16.9 Hz, H-2), 2.18 (1H, d, *J* = 16.9 Hz, H-2), 5.90 (1H, t, *J* = 1.3 Hz, H-4), 5.83 (1H, d, *J* = 15.9 Hz, H-7), 6.37 (1H, d, *J* = 15.9 Hz, H-8), 5.70 (1H, t, *J* = 6.7 Hz, H-10), 4.51 (1H, dd, *J* = 12.8, 6.7 Hz, H-11), 4.37 (1H, dd, *J* = 12.8, 6.7 Hz, H-11), 1.05 (3H, s, H-12), 1.01 (3H, s, H-13), 1.91 (3H, d, *J* = 1.3 Hz, H-14), 1.83 (3H, br s, H-15), 4.29 (1H, d, *J* = 7.8 Hz, H-1'), 3.18 (1H, dd, *J* = 9.1, 7.8 Hz, H-2'), 3.34 (1H, dd, *J* = 9.6, 9.1 Hz, H-3'), 3.28 (1H, t, *J* = 9.6 Hz, H-4'), 3.25 (1H, m, H-5'), 3.87 (1H, dd, *J* = 11.9, 2.0 Hz, H-6'), 3.67 (1H, dd, *J* = 11.9, 5.4 Hz, H-6'); ^{13}C NMR (CD_3OD, 125 MHz) δ 42.7 (C-1), 50.7 (C-2), 201.2 (C-3), 127.1 (C-4), 167.2 (C-5), 80.4 (C-6), 130.0 (C-7), 135.7 (C-8), 137.5 (C-9), 129.4 (C-10), 66.4 (C-11), 24.6 (C-12), 23.5 (C-13), 19.6 (C-14), 13.0 (C-15), 103.2 (C-1'), 75.1 (C-2'), 78.1 (C-3'), 71.7 (C-4'), 78.0 (C-5'), 62.8 (C-6').

References

1. Kai, H.; Baba, M.; Okuyama, T. Two new megastigmanes from the leaves of *Cucumis sativus*. *Chem. Pharm. Bull.* **2007**, *55*, 133–136.
2. Mohamed, K.M.; Mohamed, M.H.; Ohtani, K.; Kasai, R.; Yamasaki, K. Megastigmane glycosides from seeds of *Trifolium alexandrinum*. *Phytochemistry* **1999**, *50*, 859–862.
3. Fröhlich, O.; Schreier, P. Additional volatile constituents of carambola (*Averrhoa carambola* L.) fruit. *Flavour Fragr. J.* **1989**, *4*, 177–184.
4. Pabst, A.; Barron, D.; Sémon, E.; Schreier, P. 4-Oxo-*β*-ionol and linalool glycosides from raspberry fruits. *Phytochemistry* **1992**, *31*, 4187–4190.
5. Wang, Y.; Zhu, H.; Wang, D.; Cheng, R.; Yang, C.; Xu, M.; Zhang, Y. A new phloroglucinol glucoside from the whole plants of *Glochidion eriocarpum*. *Bull. Korean Chem. Soc.* **2014**, *35*, 631–634.
6. Otsuka, H.; Takeda, Y.; Yamasaki, K.; Takeda, Y. Structural elucidation of dendranthemosides A and B: two new *β*-ionone glucosides from *Dendranthema shiwogiku*. *Planta Med.* **1992**, *58*, 373–375.
7. Chen, B.; Li, B.G.; Zhang, G.L. Glycosides from *Erigeron breviscapus*. *Acta Bot. Sin.* **2002**, *44*, 344–348.
8. Murakami, T.; Kishi, A.; Yoshikawa, M. Medicinal flowers. IV. Marigold. (2): structures of new ionone and sesquiterpene glycosides from Egyptian *Calendula officinalis*. *Chem. Pharm. Bull.* **2001**, *49*, 974–978.
9. Wang, X.; Hu, Y.; Yuan, D.; Fu, H. Chemical constituents from the flowers of *Rhododendron molle* G. Don. *J. Chin. Pharm. Sci.* **2014**, *23*, 94–98.

10. Kato-Noguchi, H.; Tanaka, Y.; Murakami, T.; Yamamura, S.; Fujihara, S. Isolation and identification of an allelopathic substance from peel of *Citrus junos*. *Phytochemistry* **2002**, *61*, 849–853.
11. Ramos, M.D.R.; Jerz, G.; Villanueva, S, López-Dellamary, F.; Waibel, R.; Winterhalter, P. Two glucosylated abscisic acid derivates from avocado seeds (*Persea americana* Mill. Lauraceae cv. Hass). *Phytochemistry* **2004**, *65*, 955–962.
12. Yamauchi, S.; Abe, F.; Kinoshita, Y. Germination inhibitors in *Citrus unshu* fruit peelings: (+)-abscisyl *β*-D-glucopyranoside. *Biosci. Biotechnol. Biochem.* **1995**, *59*, 1968.
13. Gunawardena, D.C.; Jayasinghe, L.; Fujimoto, Y. Phytotoxic constituents of the fruits of *Averrhoa carambola*. Chem. Nat. Comp. **2015**, *51*, 532–533.
